# The prognostic value of miRNA-18a-5p in clear cell renal cell carcinoma and its function via the miRNA-18a-5p/HIF1A/PVT1 pathway

**DOI:** 10.7150/jca.36822

**Published:** 2020-02-20

**Authors:** Huan Wang, Zhong-Yi Li, Zu-Hao Xu, Yuan-Lei Chen, Ze-Yi Lu, Dan-Yang Shen, Jie-Yang Lu, Qi-Ming Zheng, Li-Ya Wang, Li-Wei Xu, Ding-Wei Xue, Hai-Yang Wu, Li-Qun Xia, Gong-Hui Li

**Affiliations:** 1 Department of Urology, Sir Run Run Shaw Hospital, Zhejiang University School of Medicine, Hangzhou, China, 310016; 2 Department of Urology, the Second Affiliated Hospital of Zhejiang University School of Medicine, Hangzhou, China, 310016

**Keywords:** clear cell renal cell carcinoma, prognosis, biomarker, microarray, molecular pathways

## Abstract

**Purpose** Clear cell renal cell carcinoma(ccRCC) is the most common type of renal cell carcinoma. While it is curable when detected at an early stage, some patients presented with advanced disease have poor prognosis. We aimed to identify key genes and miRNAs associated with clinical prognosis in ccRCC.

**Methods** The microarray datasets were obtained from the Gene Expression Omnibus database. Differentially expressed genes (DEGs) and differentially expressed miRNAs (DEMs) were analyzed by using GEO2R. Then, Functional enrichment analysis was performed using the DAVID. A retrospective series of 254 ccRCC patients with complete clinical information was included in this study. Kaplan-Meier analysis and multivariate cox regression analysis were used for prognostic analysis. Wound healing assay and transwell assay were designed to evaluate the migration and invasion ability of ccRCC cell lines.

**Results** miRNA-18a was identified to be related with prognosis of ccRCC by using Kaplan-Meier analysis and multivariate cox regression analysis demonstrated that the prognostic value of miRNA-18a was independent of clinical features. Further studies showed that up-regulation of miRNA-18a had a positive effect on migration and invasion of ccRCC cells. The target gene (HIF1A) of the miRNA-18a was predicted by using the miRPathDB database. The transcription factors of DEGs were identified by using the i-cisTarget. Luckily, HIF1A was found to be one of the transcription factors of DEGs. Among these DEGs, PVT1 may be regulated by HIF1A and be related with prognosis of ccRCC. Finally, validation of miRNA18a/HIF1A/PVT1 pathway was checked via reverse transcription-polymerase chain reaction (RT-PCR) assay in both cell lines and clinical tumor samples.

**Conclusion** Our research revealed that miRNA18a/HIF1A/PVT1 pathway might play a crucial role in ccRCC progression, providing novel insights into understanding of ccRCC molecular mechanisms. Importantly, miRNA-18a could serve as a potential diagnostic biomarker and therapeutic targets for ccRCC patients.

## Introduction

Renal cell carcinoma (RCC) accounts for approximately 3.8% of all cancer incidences and 2.5% of all cancer deaths. In recent years, the incidence of RCC, has been rising by 0.6% per year^1^, ^2^. Clear cell renal cell carcinoma(ccRCC), associated with mutation of von Hippel-Lindau gene(VHL), is the most common subtype of RCC^3^. A quarter of the patients present with advanced disease at the first time of diagnosis, including locally invasive or metastatic renal cell carcinoma. Moreover, a third of the patients who undergo resection of localized disease will have a recurrence^3^, ^4^. Thus, there is an urgent need to investigate the molecular mechanisms of the ccRCC tumorigenesis and metastasis for early diagnosis and treatment.

MicroRNAs(miRNA) are a group of non-coding small RNAs which negatively regulate the genes expression by binding to untranslated region (UTR) of target mRNA^5^. In the last decade, there were a large number of published studies that described the close link between miRNA and cancers. miRNA may act as oncogenes or suppressors in tumor development and progression through controlling expression of their target mRNA to influence the tumor cells proliferation, differentiation, migration, invasion or apoptosis. In addition, tumor microRNA profiles can define relevant subtypes, patient survival, and treatment response 6, 7. Furthermore, an increasing number of miRNAs have already been implicated in RCC such as miR-663a, miR-425-5p, miR-224 and miR-384 8-11.

HIF1A, a key transcription factor regulating cellular and homeostatic response to hypoxia, has been shown to contribute to angiogenesis, glucose metabolism, cell growth, metastasis, and apoptosis in many tumor types^12^. However, HIF1A might act as a tumor suppressor in the context of renal carcinoma^13^, ^14^. PVT1 has been identified a candidate oncogene associate with various tumors including non-small-cell lung cancer^15^, breast cancers^16^, colon cancer^17^, acute myeloid leukemia^18^.

**Figure 1:**
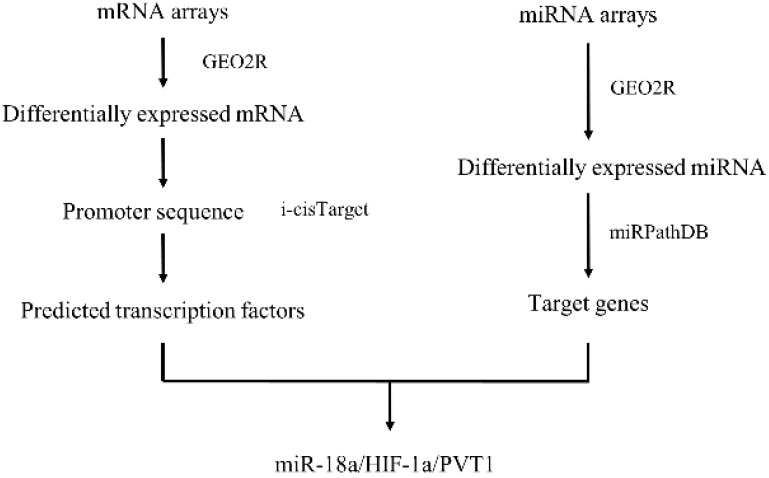
Work flow

With the advances made in high-throughput experimental technologies, such technologies are able to provide novel strategies to systematically explore the biological mechanisms of miRNA in ccRCC^19^, ^20^. In this study, we have integrated data from GEO database to explore the differently expressed genes (DEGs) and differently expressed miRNAs (DEMs) between normal tissues and tumors. Then we came up with a network and pathway-based approach and found miRNA-18a/HIF1A/PVT1 pathway which might exert potentially important roles in the development and metastasis of ccRCC. Importantly, the relationship between miRNA-18a/HIF1A/PVT1 expression level and overall survival was determined by the Cancer Genome Atlas (TCGA) data and the prognostic value of miRNA-18a was independent of clinical pathological variables. Finally, the expression of miRNA-18a/HIF1A/PVT1 was validated in both cell lines and clinical tumor samples by utilizing quantitative polymerase chain reaction (qPCR) analysis, which could be used as potential biomarkers for early diagnosis and target for treatment. Figure 1 shows the work flow of our study.

## Materials and Methods

### Cell culture and transient transfection

ACHN, OSRC-2, HK-2, Caki-1, 786-O and A498 cell lines were used for the designed experiments within 15 passages. All cells were cultured in Dulbecco's Modified Eagle's Med (Invitrogen, Grand Island, NY, USA) supplemented with 10% FBS (v/v), penicillin (25 units/ml), streptomycin (25 g/ml), 1%L-glutamine and 10% fetal bovine serum (FBS) under a condition of 5% (v/v) CO2 humidified incubator at 37℃. RCC cell lines 786-O and A498 were transfected with miRNA-18a and miRNA inhibitor using RNAiMAX (Invitrogen, Thermo Fisher Scientific, USA) following the manufacturer's instructions. MiRNA-18a, its inhibitor and normal control were purchased from RiboBio (Guangzhou, China). The transfected cells were harvested at 48 h after transfection.

### Wound healing assay and cell invasion assay

Wound healing assay was designed to evaluate the mobility of 786O and A498 cells. After transfecting for 24 h, the cells were scratch perpendicularly with a sterile 200 μl pipette tip so as to draw a straight wound. Afterwards, PBS was used to wash off the removed cells and the left cells was cultured in serum free medium for another 24 hours. The width of the scratch was measured.

RCC cells invasion ability was determined using the transwell assay. Cells (1 × 10^5) with 2% FBS media 100 ul were then placed into the upper chamber of transwell plates (8μm) with membranes precoated with 300 μg/ml Matrigel matrix (BD Corning, Corning NY, USA). Then 600 μl 10% FBS media was placed in the lower chambers for incubation at 37 °C in 5% (v/v) CO_ 2_ incubator for 24 h. Cells were observed under a microscope after staining with 1% crystal violet and the number of cells were counted in three randomly selected fields per 100× magnification field of view.

### Microarray analysis

The gene expression profiles of GSE66270^21^, GSE53757^22^, and miRNA expression profiles of GSE12105^23^ and GSE23085^24^ were obtained from National Center of Biotechnology Information (NCBI) GEO database (GEO, http://www.ncbi.nlm.nih.gov/geo/). The mRNA profiles were provided on GPL570(Affymetrix Human Genome U133 Plus 2.0 Array) while miRNA profiles were on the platforms GPL6955 (Agilent-016436 Human miRNA Microarray 1.0) and GPL10415(LC_MRA-1001_miRHuman_13.0_090309). The detailed information of these datasets was shown in Table 1.

### Differentially expressed miRNA and genes analysis

The raw data was analyzing by using GEO2R (https://www.ncbi.nlm.nih.gov/geo/geo2r/). Up-regulated and down-regulated miRNA and genes were identified between ccRCC and normal controls. A classical criteria of t test was used to identify DEGs with a change ≥2 fold and defined a P-value cutoff <0.05 to be statistically significant. Gene protein expression in ccRCC samples and normal tissues was determined by the human protein atlas (www.proteinatlas.org).

### Functional enrichment analysis

Database for Annotation Visualization and Integrated Discovery (DAVID, https://david.ncifcrf.gov/) is widely used to perform the Gene ontology analysis (GO) and Kyoto Encyclopedia of Genes and Genomes (KEGG) analysis^25^, ^26^. The human genome was chosen as the background list and human was used as the species. P<0.05 was considered statistically significant. GO enrichment analysis was a useful way to analyze biological process (BP), molecular functions (MF), and cellular components (CC) of genes. KEGG pathway enrichment analysis was used to verify the potential functional and metabolic pathways associated with DEGs.

### Quantitative real-time PCR (qRT-PCR)

Total RNA was extracted from patient tissues using TRIzol reagent (Invitrogen, USA) and was stored at -80 °C until use. Reverse Transcription System (Promega) was used for cDNA synthesis according to the protocol provided by manufacturer. The mRNA expression levels of hub genes were measured by quantitative Real time-PCR using the ABI PRISM 7500 Sequence Detector System (Applied Biosystems, USA), and was normalized to an internal standard (glyceraldehyde-3-phosphate dehydrogenase, GAPDH). For detection of miR-18a, miRNA was isolated from tissues or cells using miPure Cell/ Tissue miRNA kit (Vazyme, China), following manufacturer's instructions. cDNA was synthesized using miRNA 1st strand cDNA synthesis kit (Vazyme, China) and quantified by quantitative real-time PCR analysis experiments using miRNA universal SYBR qPCR Master Mix (Vazyme, China), according to manufacturer's instructions. U6 was used as an internal control. PCR primer used were as follows: U6-F: 5'-CTCGCTTCGGCAGCACA-3'; U6-R: 5'-AACGCTTCACGAATTTGCGT-3'; miR-18a-F: 5'-TCGCC TAAGGTGCATCTAGTGC-3'; miR-18a-R: 5'-CTCAACTGGTGTCGTGGAGTCGGC-3'; PVT1-F: 5'- GCTGTGGCTGAATGCCTCAT - 3'; PVT1-R: 5' - TTCACCAGGAAGAGTCGGGG - 3'; GAPDH-F: 5'-GGAGTCAACGGATTTGGT-3'; GAPDH-R: 5'-GTGATGGGATTTCCATTGAT-3'.

### Western blot analysis

Western blot has been described before^27^. Briefly, cells were placed on ice and washed three times with cold PBS. Proteins were extracted with RIPA buffer supplemented with 1% protease inhibitor cocktail. The protein concentration was determined with the BCA protein assay kit (Thermo Fisher Scientific). Then, the proteins were denatured at 100ºC for 15min and 30ug protein was loaded per well on 10% SDS-PAGE gel and transferred onto PVDF membrane (Thermo Fisher Scientific). Next, membranes were washed with TBST, blocked with 5% skimmed milk and incubated with antibodies against GAPDH (1:1000, sc-202525, Santa Cruz, CA, USA) and HIF1A (1:1000, ab16066, Abcam, USA). Corresponding HRP‐conjugated secondary antibodies were used against each of the primary antibody. Proteins were detected using the chemiluminescent detection reagents and the gray value was quantified by Image J software.

### Clinical samples

Tumor samples and paired adjacent non-cancerous tissues were obtained from ccRCC patients subjected to nephrectomy in the Sir Run Run Hospital of Zhejiang University who were diagnosed with ccRCC by more than two pathologists.

### Survival analysis with TCGA data

The miR-18a expression data and clinical information of ccRCC patients were obtained from TCGA database. After excluding the samples without complete information, a total of 254 ccRCC patients were enrolled in our research to analyze the relationship of miR-18a with overall survival and pathologic stage.

Similarly, to clarify the prognosis function of HIF1A and PVT1, we collected another cohort from the TCGA and The Genotype-Tissue Expression (GTEx) including 517 ccRCC patients. The Kaplan-Meier analysis was performed on the GEPIA^28^ (gepia2.cancer-pku.cn). The patient's basic information is provided in Supplementary Table 2.

### Statistical analysis

Data are presented as mean ± SEM. All experiments were performed in at least triplicate. The statistical significance between the two measurements is determined by the following equation Two-tailed unpaired Student's t-test and data were analyzed using the Prism 7.0 statistical program (GraphPad Software). P < 0.05 was considered statistically significant.

## Results

### Identification of DEGs and DEMs from mRNA and microRNA microarrays in ccRCC

**Figure 2:**
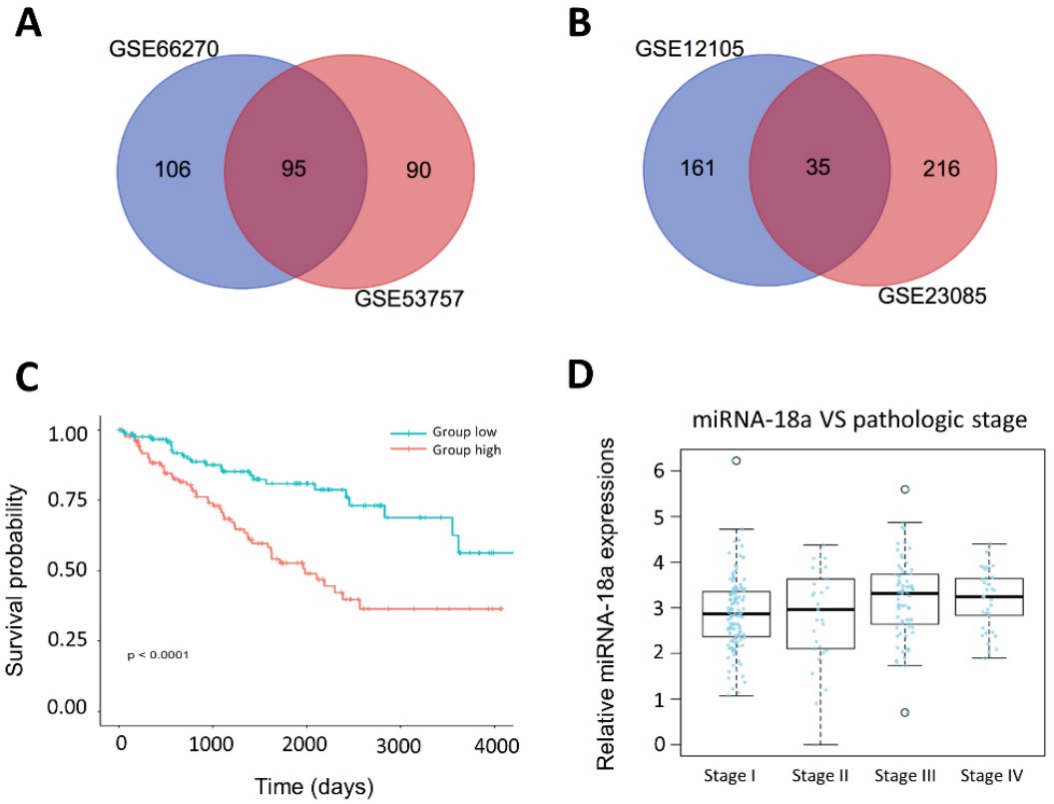
** Identification of prognosis-related miRNA from microarray. (A& B)** The result of Venn diagram. The cross areas represented the commonly changed miRNA or genes in the two different microarrays; **(C)** The relationship between the overall survival and the expression of miR-18a in ccRCC (n=254, P<0.0001); **(D)** The relationship between the pathologic stage and expression of miR-18a in ccRCC(n=254).

We obtained mRNA datasets (GSE66270, GSE53757) and miRNA datasets (GSE12105, GSE23085) from GEO database, a public functional genomics database that provides gene expression profiles (Table 1 and supplementary Table 1). Firstly, we aimed to obtain top 250 different expressed miRNAs and genes by GEO2R with the criteria P<0.05 and fold control (FC) ≥2. Then, we identified 95 DEGs and 35 DEMs by integrating above datasets. Venn diagrams were presented in Fig.2A and 2B.

### miRNA-18a was identified as a prognostic biomarker for ccRCC patients

We analyzed clinical information of 254 ccRCC patients and corresponding miRNA expression profiles from the TCGA. The characteristics of patients were showed in Table 2. Among 35 different expressed miRNAs, miRNA-18a was found to be significantly associated with overall survival of ccRCC patients. Kaplan-Meier survival curves showed that patients with the high-expression of miRNA-18a had significantly poorer survival (Fig.2C). Moreover, we found that the patients presented with stage III and IV had higher expression of miRNA-18a than the stage I and II(Fig.2D). Besides, a multivariate cox regression analysis was performed to investigate whether the prognostic value of miRNA-18a was independent of other clinical risk factors such as age, gender, tumor stage and metastasis. As is shown in Table 3, the hazard ratio (HZ) of miRNA-18a is 1.953 (P=0.005) which is higher than tumor stage, HZ=1.761(P<0.001).

These results indicated that miRNA-18a was likely to be a potential prognostic biomarker for ccRCC.

### GO term and KEGG pathway enrichment analysis of miRNA-18a

**Figure 3:**
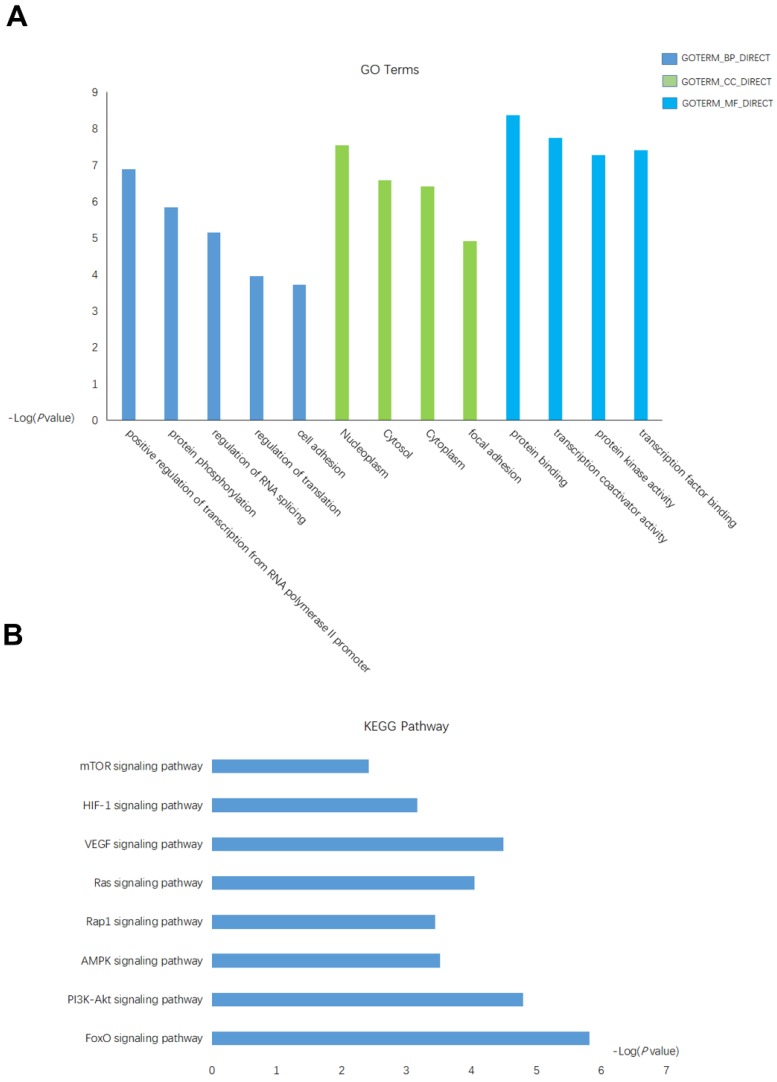
** The functional analysis of targets gene of miRNA-18a. (A)** Gene Ontology (GO) analysis and significant enriched GO terms related with miRNA-18a. **(B)** Significantly enriched KEGG pathway terms related with miRNA-18a.

The putative targets gene of miRNA-18a were predicted by using database miRPathDB^29^. A total of 128 target genes were identified as potentially regulated by the miRNA-18a. Then, we performed the Gene Ontology (GO) and Kyoto Encylopedia of Genes and Genomes (KEGG) to investigate the specific biological functions of these genes by utilizing the biological tool Database for Annotation, Visualization, and Integrated Discovery (DAVID). Enrichment of these genes were found in several GO biological processes, significantly in positive regulation of transcription from RNA polymerase II promoter, regulation of RNA splicing, regulation of translation, nucleoplasm, protein binding (Fig.3A), which indicated some of these genes may function as transcription factor (TF) that regulate the transcription of downstream genes. Further KEGG enrichment analysis revealed an overrepresentation involved in the key pathway linked to the tumor-promoting function such as: mTOR signaling pathway, HIF-1 signaling pathway, VEGF signaling pathway(Fig.3B).

Taken together, these results indicated a potentially important role of miRNA-18a in the progression of ccRCC.

### miRNA-18a/HIF1A/PVT1 may play crucial role in prognosis of ccRCC

**Figure 4:**
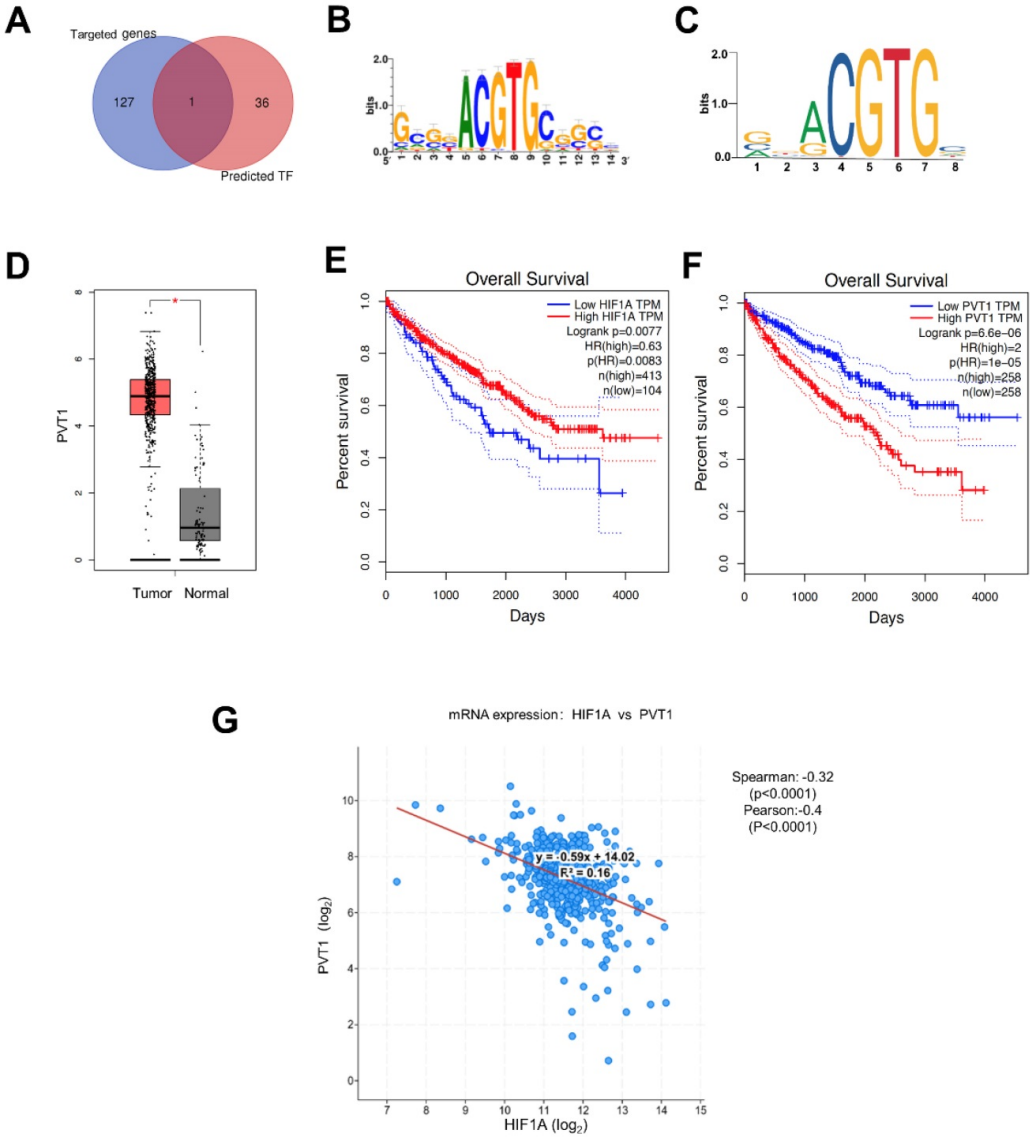
** The potential downstream pathway of miRNA-18a. (A)** Identification of HIF1A as a target gene of miRNA and a transcription factor (TF) of DEGs in ccRCC; **(B & C)** HIF1A motif sequence predicted by i-cisTarget and JASPAR; **(D)** The expression of PVT1 in ccRCC; **(E & F)** The relationship between the overall survival and expression of HIF1A and PVT1. HR, Hazard ratio; **(G)** Analysis of co-expression: HIF1a and PVT1 by cBio Cancer Genomics Portal, Spearman=-0.32 and Pearson=-0.40.

Most of genes contain the sequence-specific DNA to bind TF that play a crucial role as a regulator for the genes expression^30^. In this way, we hypothesize that miRNA-18a may regulate the mRNA expression of DEGs through the TFs. So, we utilized i-cisTarget, an integrative genomics method for the prediction of regulatory features and cis-regulatory modules, to detect some TFs that may regulate DEGs according to the their core promoters^31^. Finally, we found 37 potential TFs of DEGs and analyzed the correlations with the target genes of miRNA-18 (Fig.4A). Luckily, HIF1A, identified as a target gene of miRNA-18a, could also regulate the expression of PVT1 as a TF^32^-^34^. JASPAR (http://jaspar.genereg.net/) and i-cisTarget (https://gbiomed.kuleuven.be/apps/lcb/i-cisTarget/) database were used to search for potential HIF1A response elements (HIF1AREs) on the promoter region of PVT1 (Fig.4B and 4C). PVT1 is one of the DEGs, which is elevated in ccRCC (Fig.4D). Moreover, the clinical information of 517 ccRCC patients in TCGA showed that patients with low expression of HIF1A and high expression of PVT1 have poorer survival (Fig.4E and 4F). To determine the relationship between HIF1a and PVT1, we conducted the Pearson and spearman correlational analysis from TCGA (Fig.4G). It was apparent that the expression of HIF1A had a significantly negative correlation with PVT1 (Pearson=-0.28, spearman= -0.40).

**Figure 5:**
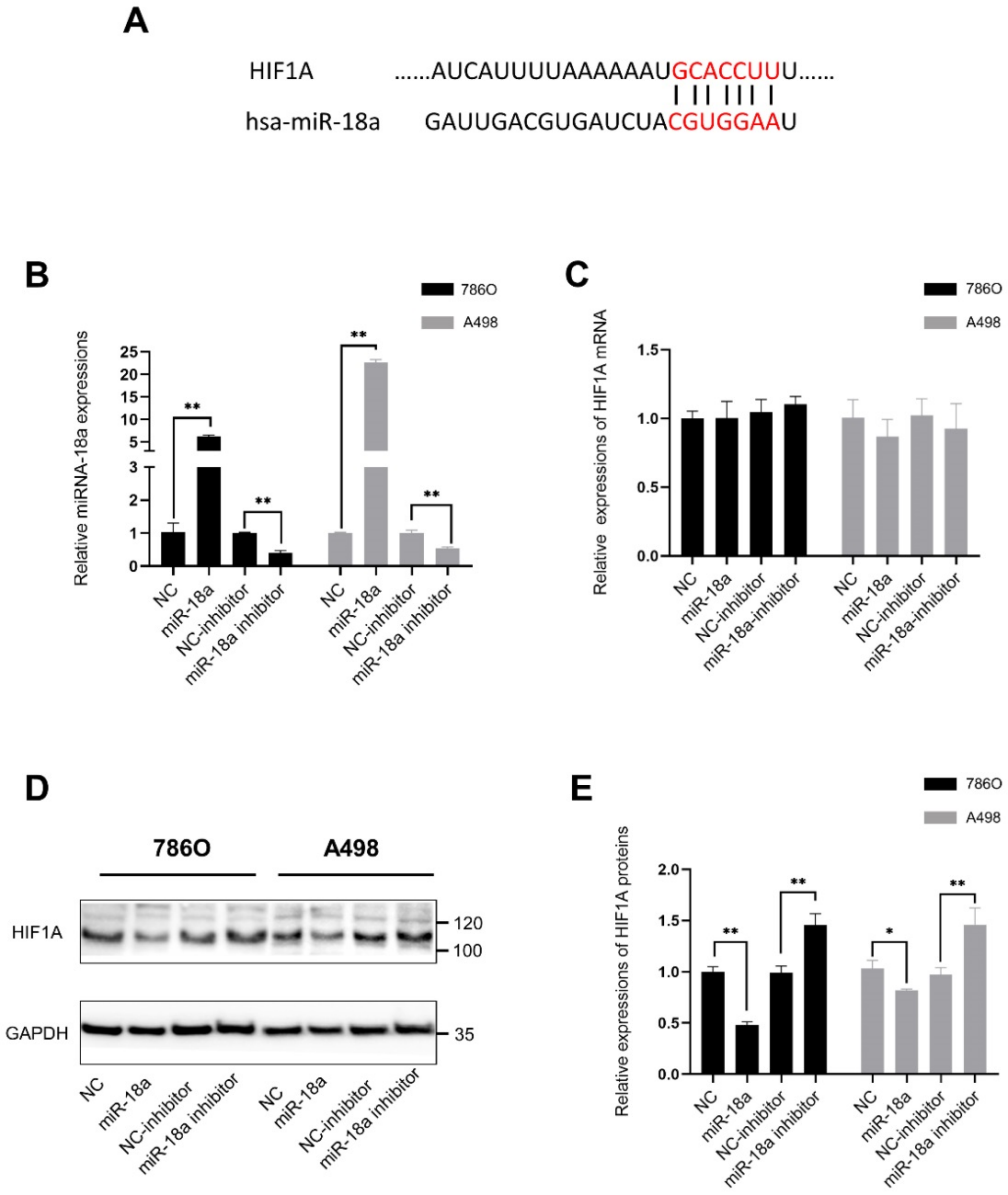
** HIF1A is the target gene of miR-18a. (A)** Duplex formation between the 3'-Untranslated Region (UTR) of HIF 1A and miR-18a; **(B)** The relative expression levels of miR-18a after transfection with miR-18a mimic, inhibitor, negative control (NC) and NC inhibitor in 786-O and A498 cell lines; **(C)** The relative expression levels of HIF1A mRNA after transfection with miR-18a mimic, inhibitor, NC and NC inhibitor in 786-O and A498 cell lines; **(D&E)** Western blot analysis showing the protein expression levels of HIF-1A in the A498 and 786O cell lines transfected with the miR-18a mimic, inhibitor, NC and NC inhibitor.

Besides, the predicted duplex formation between the 3'-UTR of HIF-1 α and miRNA-18a was shown in Fig. 5A which had also been proved by previous researches 34-36. In our study, we also compared the mRNA and protein levels of HIF1A after overexpressing miRNA-18a or inhibiting miRNA-18a.To validate the transfection efficiency in 786O and A498, qPCR assay was performed. The expression of miRNA-18a was significantly elevated after miRNA-18a overexpression, while it was decreased after miRNA-18a inhibition (Fig. 5B). Although HIF1A mRNA level was not significantly changed neither by overexpressing nor by inhibiting miRNA-18a(Fig.5C), HIF1A protein reduction was observed after overexpressing the miRNA-18a while the HIF1A protein was increased after inhibiting the miRNA-18a (Fig.5D and E). These results indicated that miRNA-18a regulated HIF-1α might through the post-transcriptional control.

In summary, these results suggested that miRNA-18a/HIF1A/PVT1 pathway might play a crucial role in ccRCC.

### miRNA-18a regulated the migration and invasion in cell lines

**Figure 6:**
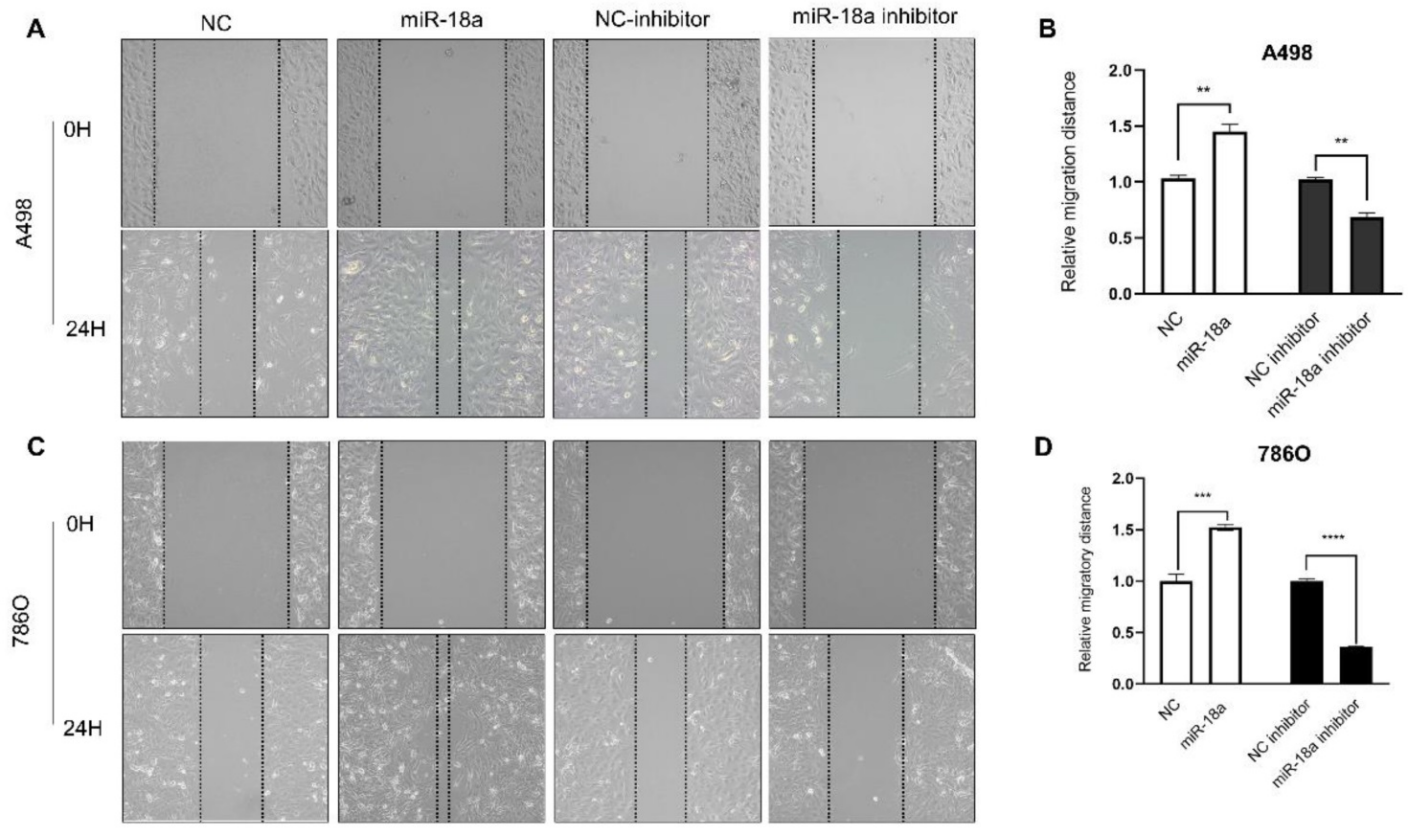
** Result of wound healing assay. (A&C)** The wound healing images in 786-O and A498 cells after transfection with miR-18a mimic, inhibitor, negative control (NC) and NC inhibitor. **(B&D)** The relative migratory distances of 786-O and A498 cells. **P < 0.01, ***P < 0.001, ****P<0.0001

The migration ability of ccRCC cells after miRNA-18a manipulation was estimated by the wound healing assay. As shown in Fig 6, the migration ability of miRNA-18a inhibitor transfected cells reduced by 33.33% (A498, **P<0.01), 63.77% (786O, ****P<0.0001) compared with negative control inhibitor (NC-inhibitor) while the migration ability of miRNA-18a mimic transfected cells increased by 42.13%(A498, **P<0.01), 52.39%(786O, ***P<0.001) compared with negative control(NC).

**Figure 7:**
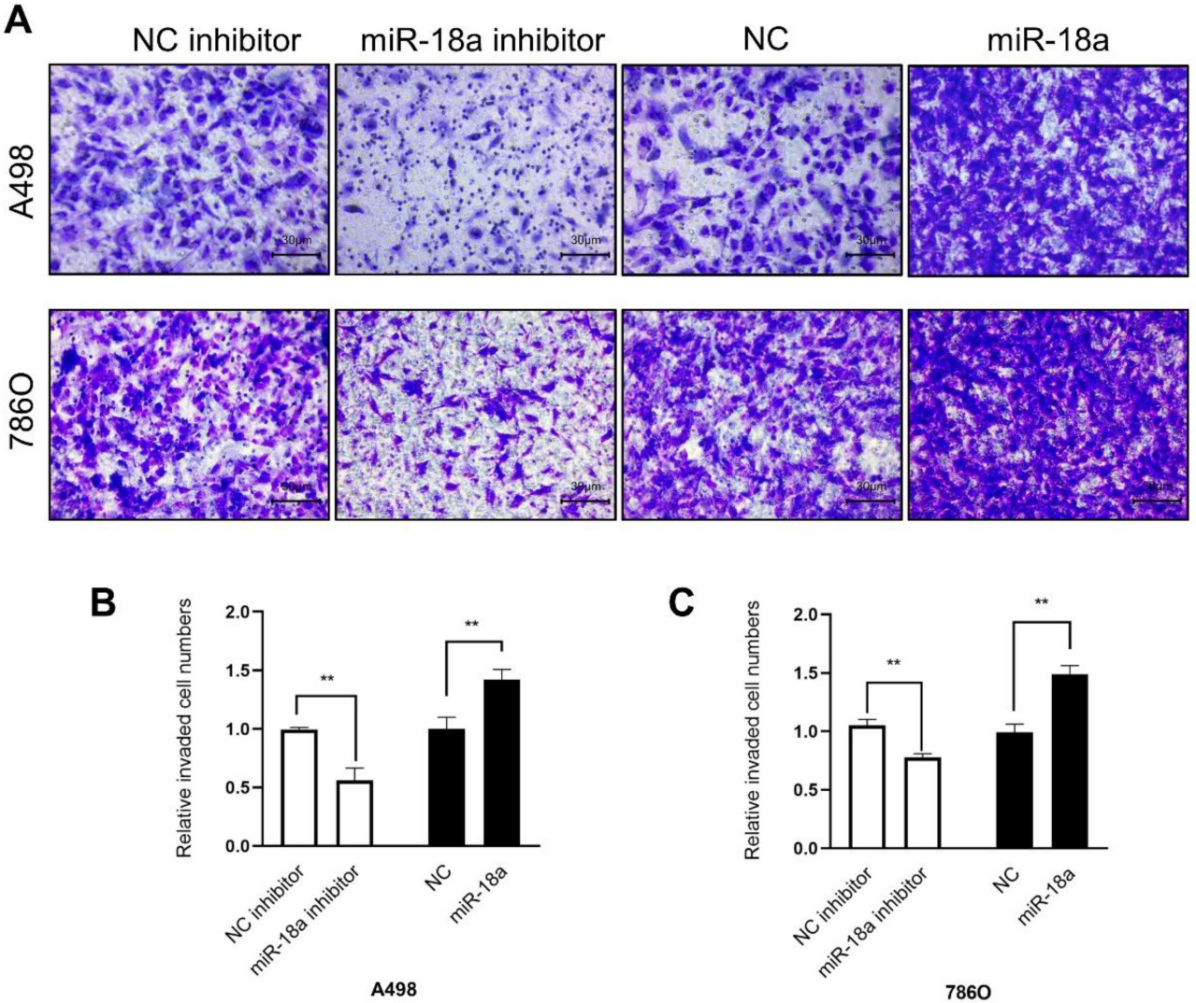
** Result of transwell assay. (A)**The invasive images of 786-O and A498 cells after transfection with miR-18a mimic, inhibitor, negative control (NC) and NC inhibitor. **(B&C)** The relative number of the invasive 786-O and A498 cells. **P < 0.01, ***P < 0.001.

The invasion ability was determined by the transwell assay. As shown in Fig 7, the invasion ability of down-regulation miRNA-18a reduced by 43.84% (A498, **P<0.01) , 22.09% (786O, **P<0.01) compared with NC-inhibitor and the invasion ability of up-regulation miRNA-18a increased by 42.08% (A498, **P<0.01),49.02% (786O, **P<0.01) compared with NC.

In conclusion, these results indicated that miRNA-18a promoted the ccRCC cells migration and invasion.

### Validation the expression of miRNA-18a and its target genes in cell lines and clinical samples

**Figure 8:**
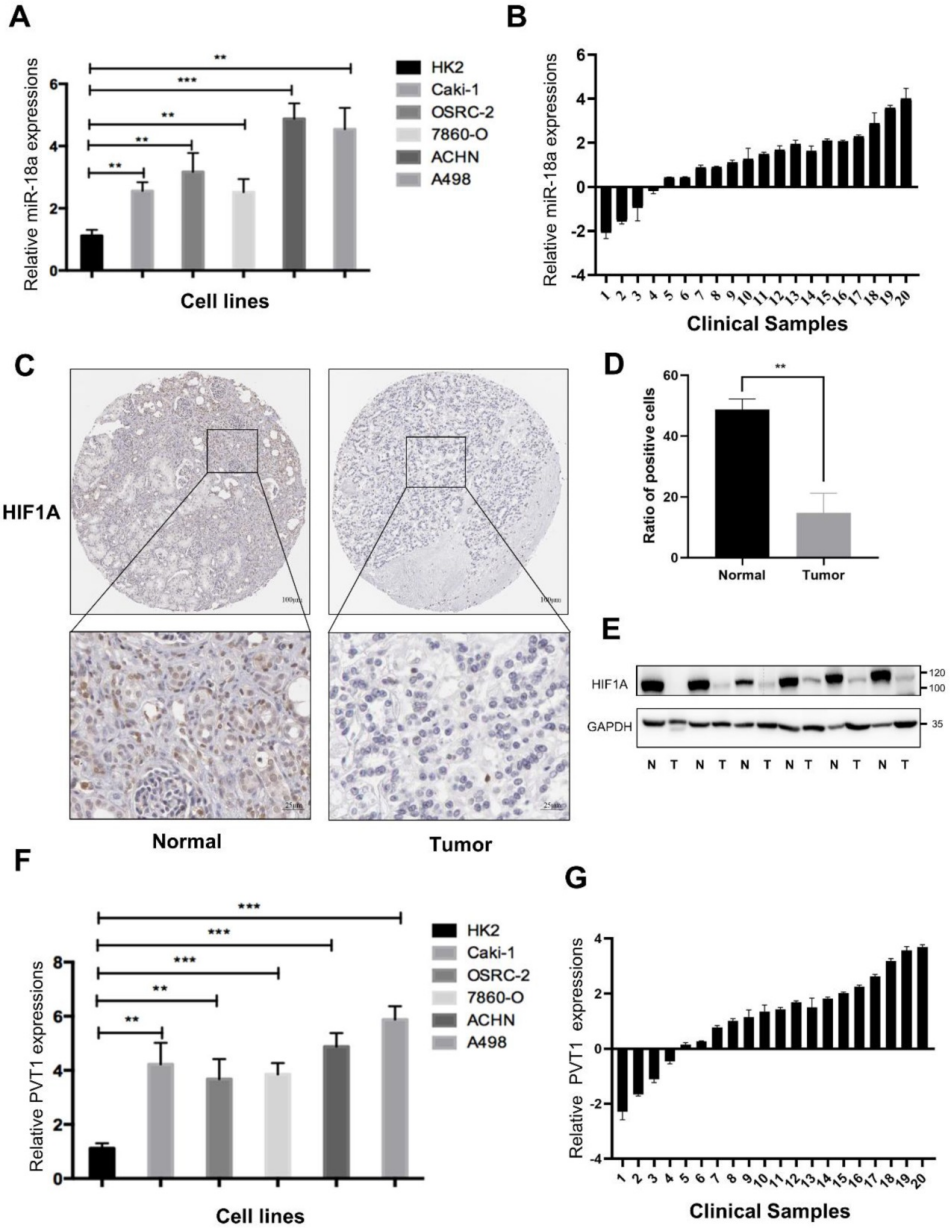
** The expression of miRNA-18a/HIF1a/PVT1 in ccRCC cell lines and clinical sample. (A)** The relative expression levels of miR-18a in ccRCC cell lines compared with normal kidney cell lines (HK2); **(B)** The relative expression of miRNA-18a in 20 paired tissues [log2(Tumor/Normal)]; **(C)** Immunohistochemistry (IHC) detection of HIF1A in normal and tumor tissues. Scale bar,Top:100μm, bottom:25μm; **(D)** Ratio of HIF1A positive cells to total cells in the tissues. The number of cells in three different field of view was counted; **(E)** The HIF1A protein expression levels were screened through Western blotting analysis in 6 paired tissues (N: normal, T: tumor); **(F)** The relative expression levels of PVT1 in ccRCC cell lines and HK2; **(G)** The relative expression of PVT1 in 20 paired tissues [log2(Tumor/Normal)].

To verify the expression of miRNA-18a and its targeted genes, we utilized RT-qPCR and found the expression of miRNA-18a was significantly elevated in 786-O, CAKI1, OSRC, ACHN and A498 compared to that in HK-2 cell (Fig.8A). Similarly, 20 cases of ccRCC tissues and corresponding normal samples from Sir Run Run Shaw Hospital were used to further demonstrate the higher expression of miRNA-18a in ccRCC samples than normal samples (Fig.8B). Moreover, the results of IHC indicated that the expression of HIF1A, a targeted gene of miRNA-18a, was lower in ccRCC than normal samples (Fig.8C and 8D). Western blotting also demonstrated HIF1A was lower in ccRCC than normal samples (Fig.8E). The high expression of PVT1 was also found in both ccRCC cell lines and clinical tumor tissues (Fig.8F and 8G).

Taken together, all these results suggested that miRNA-18a/HIF1a/PVT1 might act as a crucial pathway in ccRCC progression.

## Discussion

ccRCC, a highly metastatic tumor, is one of the most common tumor types in RCC, which may resist most adjuvant therapy such as radiotherapy, chemotherapy, immune and endocrine therapy^37^. Nowadays, anti-VEGF targeted therapy is still the first line therapy for metastatic ccRCC even though it may only bring benefit of survival for 6-15 months^38^-^40^. Thus, it is of great importance to explore the underlying mechanism of ccRCC carcinogenesis and progression to look for reliable and effective molecular targets or biomarkers for the early diagnosis, clinical treatment and predicting the prognosis of ccRCC patients after surgery.

In recent years, many attentions are paid to miRNAs, which can regulate gene expression at post-transcriptional level and play a crucial role in carcinogenesis and development of different cancers^41^-^43^. A large number of researches had indicated that abnormally expressed miRNAs could be served as an early diagnosis or prognostic markers for various cancer patients. Previous studies have reported that miRNA-18a might serve as a tumor suppressor in osteosarcoma and colorectal cancer^44^, ^45^. miRNA‑18a exhibits a protective role in CRC and osteosarcoma via inhibiting proliferation, invasion and migration by directly targeting the TBPL1 and MED27 genes respectively^46^, ^47^. Besides, the reduced expression of miRNA-18a may associate with hepatocellular carcinoma recurrence through the upregulation of a crucial oncogene, K-ras^48^-^50^. Previous studies have demonstrated that PVT1 regulated the expression of HIF1A via competing for miR-199a-5p as a competing endogenous RNA (ceRNA) in non‑small cell lung cancer^51^. Nevertheless, searching for more miRNAs targeting HIF1A might assist to better understand the pathogenesis of ccRCC.

In the present study, we obtained mRNA datasets (GSE66270, GSE53757) and miRNA datasets (GSE12105, GSE23085) from GEO database to screen the DEGs and DEMs between ccRCC and adjacent normal tissues by bioinformatics analyses. In order to establish the connection between the DEGs and DEMs, we tried to verify the target genes of DEMs by miRpathDB. Fortunately, through the analysis the TFs of the DEGs, we found HIF1A could be a bridge connecting miRNA-18a to PVT1.

In order to further enhance the understanding of the miRNA-18a/HIF1A/PVT1 pathway in the pathogenesis of ccRCC, we found that miRNA-18a was elevated in both ccRCC cell lines and clinical samples and was related with a poor overall survival in ccRCC patients by TCGA data. Unlike previous studies^36^, we noticed that miRNA-18a significantly decreases the protein expression level of HIF1A rather than mRNA. miRNAs could regulate the gene expressions not only by inducing the direct degradation of target mRNAs, but also inhibiting the translation by binding to the 3' UTR of mRNA^52^. In accordance with these results, miRNA-18a might bind to the 3'UTR of the mRNA to inhibit the translation of HIF1A in the ccRCC. Moreover, we identified that HIF1A, the targeted gene of miRNA-18a, can negatively regulate the expression of PVT1 at transcription level through network-based studies. Consistently, reduced expression of HIF1A was related with a poor clinical outcome of ccRCC and elevated expression of PVT1 was associated with a poor clinical outcome of ccRCC patients. Wound healing assay and transwell assay were used to observe the miRNA-18a effect on RCC cell lines. The results revealed that miRNA-18a promoted the migration and invasion of ccRCC cells. RT-qPCR was used to demonstrated the expression of miRNA-18a/ HIF1A / PVT1 in both ccRCC tissues and cell lines. The construction of these regulatory networks will contribute in revealing the potential mechanism of these hub genes in ccRCC initiation and progression. The limitation of our study is that the more potential mechanism of miRNA-18a in ccRCC needs to be further verified.

To sum up, results from present study indicated that the expression of miRNA-18a was increased in ccRCC. We further found that upregulation of miRNA-18a could decreased the expression of HIF1A at post-transcriptional level. In addition, Data of TCGA reveal that patients with high expression of miRNA-18a had significantly poorer overall survival and it is an independent risk factor. More importantly, the migration and invasion of RCC cells increased after overexpressing miRNA-18a while down-regulation miRNA-18a reversed the effect. These results demonstrated that miRNA-18a might function as an oncogene and prognostic biomarker via miRNA-18a/HIF1A/PVT1 pathway in ccRCC.

## Supplementary Material

Supplementary figures and tables.

## Figures and Tables

**Table 1:** Statistics of the four microarray datasets obtained from the GEO database

Dataset ID	ccRCC samples	Normal samples	Total number
GSE66270	14	14	28
GSE53757	72	72	144
GSE12105	12	12	24
GSE23085	20	20	40

**Table 2:** Patient characteristics

Variables	Case number (N=254) N% or mean (range)
**Gender**	
Male	171(67.3%)
Female	83 (32.7%)
**Age (years)**	
Male	61.80 (29-86)
Female	58.41 (26-90)
**Tumor stage**	
I	131 (51.6%)
II	28 (11.0%)
III	49 (19.3%)
IV	46 (18.1%)

**Table 3:** Multivariate Cox regression analysis of prognostic value of miR-18a for ccRCC patients

Variables	HR	95%CI of HR	*P-*value
miR-18a	1.953	1.227-3.109	=0.005
Age	1.028	1.006-1.050	=0.013
Gender	0.993	0.592-1.666	=0.978
Stage	1.761	1.302-2.381	<0.001

## Abbreviations

ccRCC: clear cell renal cell carcinoma
DEGs: different expressed genes
GEO: Gene Expression Omnibus
TCGA: The Cancer Genome Atlas
HR: hazard ratio
CI: confidence interval
IHC: Immunohistochemistry
OS: overall survival
HIF1A: hypoxia inducible factor 1 subunit alpha
PVT1: Plasmacytoma Variant Translocation 1
