# Supplementary material for: The prognostic value of miRNA-18a-5p in clear cell renal cell carcinoma and its function via the miRNA-18a-5p/HIF1A/PVT1 pathway
Source: J Cancer. 2020 Feb 20;11(10):2737–48. doi: 10.7150/jca.36822 (PMC7086242; doi:10.7150/jca.36822)
Supplement: Supplementary file 1 — Supplementary figures and tables. [file jcav11p2737s1.pdf]

Supplementary Table 1: Patient characteristics from GEO datasets

**Variables**		**GSE66270**	**GSE53757**	**GSE12105**	**GSE23085**
Gender	Male	11	-	7	15
	Female	3	-	5	5
Age(years)		64.62(45-78)	-	63（40-92）	57.75(29-78)
Stage	I	10	24	2	16
	II	1	19	1	2
	III	3	14	9	2
	IV	-	15	-	-

Supplementary Table 2: Patient characteristics from TCGA datasets

**Variables**		**Case number (N=517) or mean (range)**
Gender	Male	336
	Female	181
Age		60.64 (34-90)
Stage	I	266
	II	54
	III	121
	IV	76
